# Impact of pacing mode and different echocardiographic parameters on cardiac output (PADIAC)

**DOI:** 10.3389/fcvm.2023.1185518

**Published:** 2023-05-17

**Authors:** Hermann Blessberger, Juergen Kammler, Joerg Kellermair, Daniel Kiblboeck, Alexander Nahler, Denis Hrncic, Karim Saleh, Stefan Schwarz, Christian Reiter, Alexander Fellner, Christian Eppacher, Todd J. Sheldon, Clemens Steinwender

**Affiliations:** ^1^ Department of Cardiology, Kepler University Hospital, Linz, Austria; ^2^ Medical Faculty, Johannes Kepler University, Linz, Austria; ^3^ Department of Internal Medicine II, Paracelsus Medical University, Salzburg, Austria; ^4^ Field Clinical Organisation, Medtronic Austria GmbH, Vienna, Austria; ^5^ CRM Research, Medtronic PLC, Mounds View, MN, United States

**Keywords:** AV-synchronous pacing, stroke volume, speckle tracking strain echocardiography, diastolic left ventricular function, systolic left ventricular function, echo parameters

## Abstract

**Introduction:**

The extent of the hemodynamic benefit from AV-synchronous pacing in patients with sinus rhythm and AV block is not completely understood. Thus, we systematically investigated the association of an array of echocardiographic and epidemiological parameters with the change in cardiac output depending on the stimulation mode (AV-synchronous or AV-asynchronous pacing).

**Methods:**

Patients in sinus rhythm after previous dual chamber pacemaker implantation underwent a thorough basic echocardiographic assessment of diastolic and systolic left ventricular function, and atrial function (26 echo parameters, including novel speckle tracking strain measurements). Then, stroke volume was measured with AV-synchronous (DDD) and AV-asynchronous (VVI) pacing. Each patient represented their own control, and the sequence of stroke volume measurements was randomized.

**Results:**

In this prospective single-center study (NCT04068233, registration August 22nd 2019), we recruited 40 individuals. The stroke volume was higher in all patients when applying AV-synchronous DDD pacing [median increase 12.8 ml (16.9%), *P* < 0.001]. No echo parameter under investigation was associated with the extent of stroke volume increase in a linear regression model. Of all epidemiological variables, a history of acute myocardial infarction (AMI) was associated with an attenuated stroke volume gain in a univariate and a multivariate regression model that adjusted for confounders. A- and S-wave velocities were reduced in the AMI group.

**Discussion:**

In our cohort of patients, each subject benefited from AV-synchronous DDD pacing. No single echo parameter could predict the amount of stroke volume increase. The beneficial effect of AV-synchronous pacing on stroke volume was attenuated after prior acute myocardial infarction.

**ClinicalTrials.gov identifier** (NCT number): NCT04068233.

## GRAPHICAL ABSTRACT

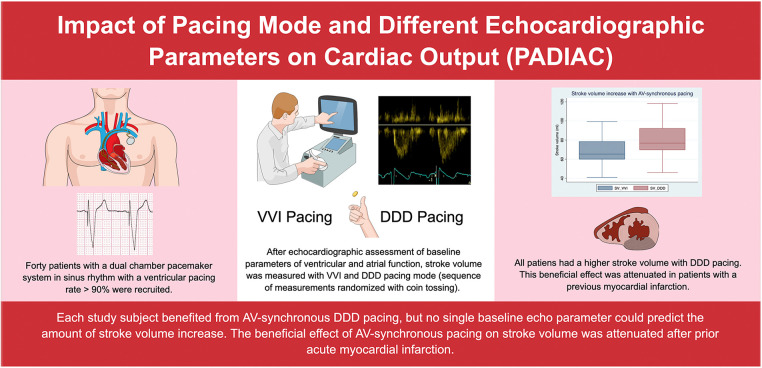

## 1. Introduction

Although no mortality benefit for atrioventricular (AV)-synchronous vs. ventricular pacing (VVI) has been shown in clinical trials, improvements in stroke volume have been demonstrated. However, it is still unclear which patient groups are most likely to receive hemodynamic benefit from AV-synchrony or to develop pacemaker syndrome (1, 2). This is currently of even greater interest as leadless cardiac pacemaker (LCP) systems are expanding the field of device therapy (3, 4). As leads and generator pockets are no longer required with this technology, many potentially dangerous complications such as lead fractures, lead endocarditis, or pocket infections can effectively be avoided (5, 6). Until recently, these benefits came with the downside of single chamber pacing. Only patients in atrial fibrillation or with an anticipated low rate of ventricular pacing were suitable candidates for leadless pacing. Recently, a leadless pacemaker capable of AV-synchronous pacing has been developed that utilizes an accelerometer to detect the atrial contraction (7), the Micra AV® (Medtronic PLC, MN, US) (8). Avoidance of two transvenous leads may be desirable in frail patients with a high-grade AV-block and a high burden of co-morbidities or in specific clinical scenarios, such as device infection or blocked venous access. It has been shown in the MARVEL 2 study that the proportion of AV-synchrony with the Micra AV® in VDD mode varied between 89.2% and 69.8% depending on body posture and level of activity (8). In a real-world setting, the rate of atrial mechanical sensing and ventricular pacing (AM-VP) – which is a surrogate for AV-synchrony–averaged 74.7% in patients who required more than 90% ventricular stimulation, reflecting the same range (9). However, it must be stressed that AV-synchrony is frequently lost if heart rates exceed 100 beats per minute or may deteriorate with change of body posture (9, 10). In selected cases, evaluation with Holter ECG and ergometry as well as sophisticated device re-programming may be required to optimize LCP function and avoid pacemaker syndrome (10). When pursuing a “patient-tailored” approach, it is essential to select and characterize the patients who will benefit most from this new technology. For this purpose, echocardiographic parameters reflecting diastolic left ventricular function were found to be useful in the past (11). Since then, progress has been made towards a better pathophysiological understanding of diastolic and systolic function based on a large body of evidence. This evolution was paralleled by the development of new echocardiographic techniques - such as speckle tracking strain analysis - and the concomitant definition of a variety of new echo parameters. The aim of this study was to systematically investigate the influence of an array of echo parameters reflecting diastolic and systolic left ventricular function and atrial function, as well as epidemiological parameters, on the change in cardiac output when comparing AV-asynchronous VVI and AV-synchronous DDD pacing in subjects with sinus rhythm.

## 2. Methods

### 2.1. Study design and patient enrollment

In this prospective, randomized, single-center, single-cohort interventional trial, we recruited patients to undergo a single echocardiographic examination with different pacing modes according to our protocol. Each patient represented their own control. We collected baseline variables (age, sex, weight, height, co-morbidities, current medication, indication and date of first pacemaker implantation, pacemaker vendor, currently programmed parameters such as pacing mode and AV-delay, and percentage of atrial and ventricular pacing). No further follow-up was performed, as participants finished the study immediately after the echo examination or after any ongoing adverse event was resolved. Approval of the study protocol by the local ethics committee of the Province of Upper Austria was sought and granted (1169/2018, issued on December 10th, 2018). The study was prospectively registered with ClinicalTrials.gov and was assigned the unique identifier NCT04068233. Subjects above the age of 18, who were scheduled for a routine device follow-up at our outpatient department, were eligible for inclusion if they were implanted with a dual chamber pacemaker system for at least six weeks and were able and willing to give informed consent. The time interval of six weeks was chosen to allow the tissue-leads interface to mature and the device parameters to stabilize after initial implantation. Patients had to be in sinus rhythm on the day of recruitment and the day of the echo examination. Upon initial device interrogation, the ventricular pacing rate had to exceed 90% due to AV-block. Moreover, the technical parameters of the pacemaker system had to be within normal ranges (atrial lead: stable pacing thresholds ≤3.0 V@0.40 ms, pacing impedances between 200 and 2,000 Ω, adequate sensing of atrial activity to allow for AV-sequential ventricular pacing; ventricular lead: stable pacing thresholds ≤2.0 V@0.40 ms, pacing impedances between 200 and 2,000 Ω; calculated battery life: more than one year). Patients were excluded if they were not in sinus rhythm or exhibited intrinsic ventricular activation on the day of the echo examination. Further exclusion criteria were relevant anatomical shunts on the atrial, ventricular, or pulmonary level, moderate or severe heart valve dysfunction (stenosis or regurgitation), presence of other medical devices that might have interacted with the pacemaker system, and pregnancy or breastfeeding in women. The exact location of the right ventricular (RV) lead was determined by analyzing the most recently available chest x-ray (posterior-anterior and lateral projections). RV lead position was classified as “mid/high septal”, “inferior septal”, “apical”, or “right ventricular outflow tract (RVOT)”.

### 2.2. Definition of endpoints

The primary endpoint was defined as the association between baseline echo parameters of left ventricular diastolic function and the change of stroke volume with different pacing modes [AV-synchronous (DDD) vs. AV-asynchronous (VVI)]. The echo parameters collected in this study are summarized in Table 1. We used stroke volume as a proxy for cardiac output, as cardiac output *per se* is heart rate dependent. The secondary endpoints were defined as the association between baseline echo parameters of (1) left ventricular systolic function and (2) left and right atrial function, as well as (3) epidemiological variables and the change of stroke volume with different pacing modes [AV-synchronous (DDD) vs. AV-asynchronous (VVI)].

**Table 1:** Echo parameters under investigation that may influence the change of stroke volume with modification of pacing mode.

Assessment of	Echo parameters	Technical considerations
LV diastolic function	• E-wave (cm/s) • A-wave (cm/s) • E/A ratio • E-wave deceleration time (ms)	PW-Doppler at the tips of the mitral valve leaflets
	• E’ septal (cm/s) • E’ lateral (cm/s) • E/E’ septal ratio • Averaged E/E’ ratio	Tissue Doppler imaging (TDI) at the medial and lateral mitral valve annulus
	• S-wave (cm/s) • D-wave (cm/s) • S/D ratio	PW-Doppler 1 cm within the pulmonary vein (was only assessed if tracing was of sufficient quality) (12)
	• Tricuspid regurgitation velocity (m/s)	CW-Doppler at the tricuspid valve
LV systolic function	• LV-EDD (mm) • LV-ESD (mm)	Parasternal long axis view
	• LV-EDV (ml) • LV-ESV (ml) • Ejection fraction (%)	Biplane Simpson's method (apical two and four-chamber view)
	• GLPSS (%)	Speckle tracking echocardiography (apical 2-, 3- and 4-chamber view).
Left atrial function	• LA volume (ml) • LAVI (ml/m^2^)	Biplane method (left atrial end-systolic volume in the apical four and two chamber view)
	• GLPSS of left atrium (LA GLPSS (%) • LA reservoir strain (LA_r_, %) • LA conduit strain (LA_cd_, %) • LA contraction strain (LA_ct_, %)	Speckle tracking echocardiography (apical four-chamber view only)
Right atrial function	• TAPSE (mm) • RA contraction excursion (RACE, mm)	M-mode tracing at the lateral tricuspid annulus, RACE = distance from mid P-wave on ECG to the nadir of M-mode tracing of the lateral tricuspid annulus during diastole

To assess these baseline parameters, the pacemaker was programmed to DDD 40/min. to allow for measurement at the patient’s intrinsic heart rate after optimization of the AV-delay. LV, left ventricular; LA, left atrial; RA, right atrial; EDD, end-diastolic diameter; ESD, end-systolic diameter; EDV, end-diastolic volume; ESV, end-systolic volume; GLPSS, global longitudinal peak systolic strain; LAVI, left atrial volume index; TAPSE, tricuspid annular plane systolic excursion.

### 2.3. Transthoracic echocardiography and device programming

Echo studies were conducted using a commercially available echo system (Epiq 7®, Philips Health Systems, Amsterdam, The Netherlands). If the pacing mode was changed, measurements started after one minute at the earliest to allow for hemodynamic adaptation. All echo examinations were performed with continuous ECG tracings and stored digitally (at least five continuous beats). One-dimensional echo parameters were averaged over three consecutive beats. Global longitudinal strain measurements were performed using a dedicated speckle tracking software (QLab, Philips, Amsterdam, The Netherlands). Left ventricular global longitudinal peak systolic strain (GLPSS) was assessed as described previously from the apical four-, two-, and three-chamber view (13, 14). Peak systolic left atrial strain was only calculated from the apical four-chamber view (15, 16). Special emphasis was put on the manual adjustment of the region of interest to fit the much thinner atrial wall. Besides the global left atrial strain (LA GLPSS), we also computed the strain during the reservoir phase (LAS_r_), the conduit phase (LAS_cd_), and the contraction phase (LAS_ct_) of the left atrium (17).

#### 2.3.1. Optimization of the AV-delay

In a first step, the AV interval was optimized by visually assessing the PW-Doppler tracing at the tips of the mitral valve leaflets. In each individual, we aimed at an AV interval that promoted ventricular pacing without (1) truncation of the A-wave or (2) E- and A-wave fusion at the baseline sinus rate. The programmed AV-delay was kept constant during the entire investigation.

#### 2.3.2. Assessment of baseline echocardiographic parameters

Parameters of left ventricular diastolic and systolic function as well as right and left atrial function were assessed applying a DDD pacing mode. Thereby, the intervention rate was set to 40 beats per minute to ensure atrial sensing and AV-synchronous ventricular pacing. Diastolic dysfunction was graded according to current guideline recommendations (grade I–III) (18). For technical considerations, see Table 1.

#### 2.3.3. Assessment of left ventricular stroke volume

Left ventricular stroke volume was measured with two different pacing modes: in (1) DDD mode with an intervention rate set to 40 beats per minute and in (2) VVI mode with the intervention rate approximately set to the intrinsic sinus rate. With the first setting, atrial sensing and AV-synchronous ventricular pacing were ensured, whereas with the second setting AV-asynchronous ventricular pacing was achieved for comparison. The sequence of pacing modes (VVI after DDD or vice versa) was randomized among the study subjects by coin tossing to account for a possible time trend bias. For echocardiographic assessment of the stroke volume, the left ventricular outflow tract (LVOT) diameter was measured in the parasternal long axis during mid-systole. A round shape of the outflow tract was assumed. Then, the LVOT volume time integral (VTI) was measured during systole with both pacing modes. The extent of mitral regurgitation was visually assessed with each pacing mode. In addition, the systolic and diastolic blood pressure were taken at the earliest one minute after changing the pacing mode using an appropriately sized blood pressure cuff. All device parameters were restored to original settings at the end of the examination.

### 2.4. Statistical methods

Continuous data are described as median, interquartile range (from the 25th to the 75th percentile). Discrete data are given as counts and percentages. Continuous variables were compared between groups using a two-sample Mann–Whitney *U* test or Kruskal–Wallis test in the case of more than two groups. A *χ*^2^ test was used for discrete data. Linear regression models were fitted to assess the impact of echo parameters on the change of left ventricular stroke volume. Clinically significant effect modifiers were accounted for by applying a multivariate regression model. As this was a hypothesis-generating study, no adjustment for multiple testing was done. Lower *p*-values indicate a stronger trend of association. All calculations were performed with the software package Intercooled STATA (release 14.0, StataCorp LP, Texas, USA).

## 3. Results

### 3.1. Characteristics of the study cohort

Between January 2019 and February 2020, we recruited 40 patients who met the predefined inclusion and exclusion criteria. The primary indication for device therapy was a complete AV-block in 31 patients (77.5%), a second-degree AV-block in 6 patients (15.0%), and a sick sinus syndrome in three patients (7.5%). Study participants received their first device a median of 5.3 years ago (IQR: 2.3–11.4 years, range: 0.3–24.4 years). RV leads were implanted at a high/mid septal position in 27 (67.5%), at an inferior septal location in one (2.5%), apically in 11 (27.5%), and in the RVOT in one (2.5%) subject. All eligible patients underwent and completed the study and were in sinus rhythm on the day of the echo examination. Two subjects reported palpitations during pacing in the VVI mode. No other adverse events or severe adverse advents occurred during the study. Baseline heart rates ranged between 41 and 92 beats per minute. For further details, see Table 2.

**Table 2:** Baseline characteristics of the study cohort.

Parameter	Median (IQR) or count (%)	Parameter	Median (IQR) or count (%)
Age (years)	76.0 (70.5–81.0)	Spironolactone	2 (5.0)
Sex (female)	9 (22.5)	Ivabradine	0 (0.0)
BMI (kg/m^2^)	27.4 (24.3–29.4)	Arterial hypertension	34 (85.0)
BP systolic (mmHg)	153 (139–166)	Diabetes mellitus type II	12 (30.0)
BP diastolic (mmHg)	86 (74–93)	Congestive heart failure	9 (22.5)
Heart rate (bpm) DDD	65 (58–75)	Hypercholesterolemia	25 (62.5)
Heart rate (bpm) VVI	64 (60–75)	Coronary heart disease	15 (37.5)
Atrial pacing (%)	39.7 (16.8–58.9)	History of myocardial infarction	6 (15.0)
Ventricular pacing (%)	99.9 (98.0–100.0)	Peripheral artery disease	2 (5.0)
Beta-blockers	20 (50.0)	History of stroke	7 (17.5)
ACE inhibitors	18 (45.0)	Chronic kidney disease (GFR < 60 ml/min/1.73 m^2^)	16 (40.0)
AT_2_-blockers	10 (25.0)	GFR stage (1/2/3/4/5)	1 (2.5)/23 (57.5)/14 (35.0)/1 (2.5)/1 (2.5)

Physiological parameters, medication, and prior medical history at the time of echo examination. IQR, interquartile range; BMI, body mass index; bpm, beats per minute; BP, blood pressure; CKD, chronic kidney disease; GFR, glomerular filtration rate (stage 1: >90, 2: 60–89, 3: 30–59, 4: 15–29, 5: <15 ml/min/1.73 m^2^).

### 3.2. AV-delay optimization, echo examinations, and change of stroke volume with pacing mode

The median heart rate at the time of the echo examination was 67 beats per minute. The median programmed optimized AV-delay was 98 ms [interquartile range (IQR): 80–135 ms, range: 60–200 ms]. All echo examinations were performed by a single investigator (HB). Image quality was judged to be good in 11 (28%), fair in 19 (48%), and poor in 10 (25%) subjects. All echo parameters could be assessed, except for tricuspid regurgitation velocity in three subjects and pulmonary vein flow signals in one study participant (see Table 3). According to randomization, stroke volume was measured first in VVI mode in 17 (42.5%) patients and first in DDD mode in 23 (57.5%) patients. The median stroke volume was 65.3 ml (IQR: 60.0–78.3 ml) with VVI and 76.8 ml (IQR: 69.8–92.2 ml) with DDD pacing mode (*p*-value for difference <0.001). In all subjects, the stroke volume was higher with DDD mode than VVI mode stimulation. The median increase with AV-synchronous DDD pacing was 12.8 ml (IQR: 5.5–16.0 ml, range: 0.1–37.7 ml, see Supplementary Figure S1). These values corresponded to a median relative increase of 16.9% (IQR: 8.5%–24.1%, range: 0.1%–58.9%). The grade of mitral valve regurgitation as visually assessed by using color Doppler imaging was lower with DDD mode than with VVI mode [DDD: median 0.5 (IQR: 0.5–1.0), VVI: 1.0 (IQR: 0.5–1.5), *p* = 0.003]. Furthermore, the median blood pressure tended to be higher with DDD pacing [VVI: 135 (123–153)/71 (64–87), DDD: 144 (132–158)/76 (66–84), *p*-values for systolic and diastolic values were 0.042 and 0.163, respectively].

**Table 3:** Baseline echo parameters of the entire cohort.

Parameter	Median (IQR)	Parameter	Median (IQR) or count (%)
E-wave (cm/s)	72.3 (58.0–93.6)	LV-EDD (mm)	46.5 (42.0–49.5)
A-wave (cm/s)	92.9 (72.0–105.0)	LV-ESD (mm)	32.0 (28.0–37.0)
E-wave deceleration time (ms)	273.0 (250.0–311.5)	LV-EDV (ml)	96.2 (68.5–126.0)
E/A ratio	0.7 (0.6–1.2)	LV-ESV (ml)	35.4 (24.8–61.0)
E’ septal	4.4 (3.8–5.3)	Septum (mm)	15 (13–17)
E’ lateral	6.5 (5.4–9.4)	Ejection fraction (%)	60.8 (50.0–63.7)
E/E’ septal ratio	16.0 (11.9–19.1)	LV GLPSS (%)	−17.3 (−20.2 to −15.4)
Averaged E/E’ ratio	13.9 (11.0–16.1)	LA volume (ml)	71.9 (54.4–92.6)
S-wave (cm/s)	60.1 (51.9–67.7)	LAVI (ml/m^2^)	36.7 (28.3–45.9)
D-wave (cm/s)	48.0 (37.9–65.5)	LA GLPSS (%)	24.3 (15.4–27.5)
S/D ratio	1.2 (0.9–1.5)	LA reservoir strain (%)	29.5 (22.5–39.0)
Tricuspid regurgitation velocity (m/s)	2.7 (2.5–2.9)	LA conduit strain (%)	−12.7 (−17.0 to −10.0)
Diastolic dysfunction (grade 0/1/2/3)	9 (22.5)/14 (35.0)/14 (35.0)/3 (7.5)	LA contraction strain (%)	−16.5 (−20.0 to −11.5)
RACE (mm)	10.5 (8.0–14.0)	TAPSE (mm)	23.5 (20.0–27.5)

Diastolic dysfunction grade 0 denotes normal diastolic function. LV, left ventricular; LA, left atrial; EDD, end-diastolic diameter; EDV, end-diastolic volume; ESD, end-systolic diameter; ESV, end-systolic volume; GLPSS, global longitudinal peak systolic strain; TAPSE, tricuspid annulus plane systolic excursion; RACE, right atrial contraction excursion.

### 3.3. Association of echo parameters and baseline variables with the increase in stroke volume

**Figure 1:**
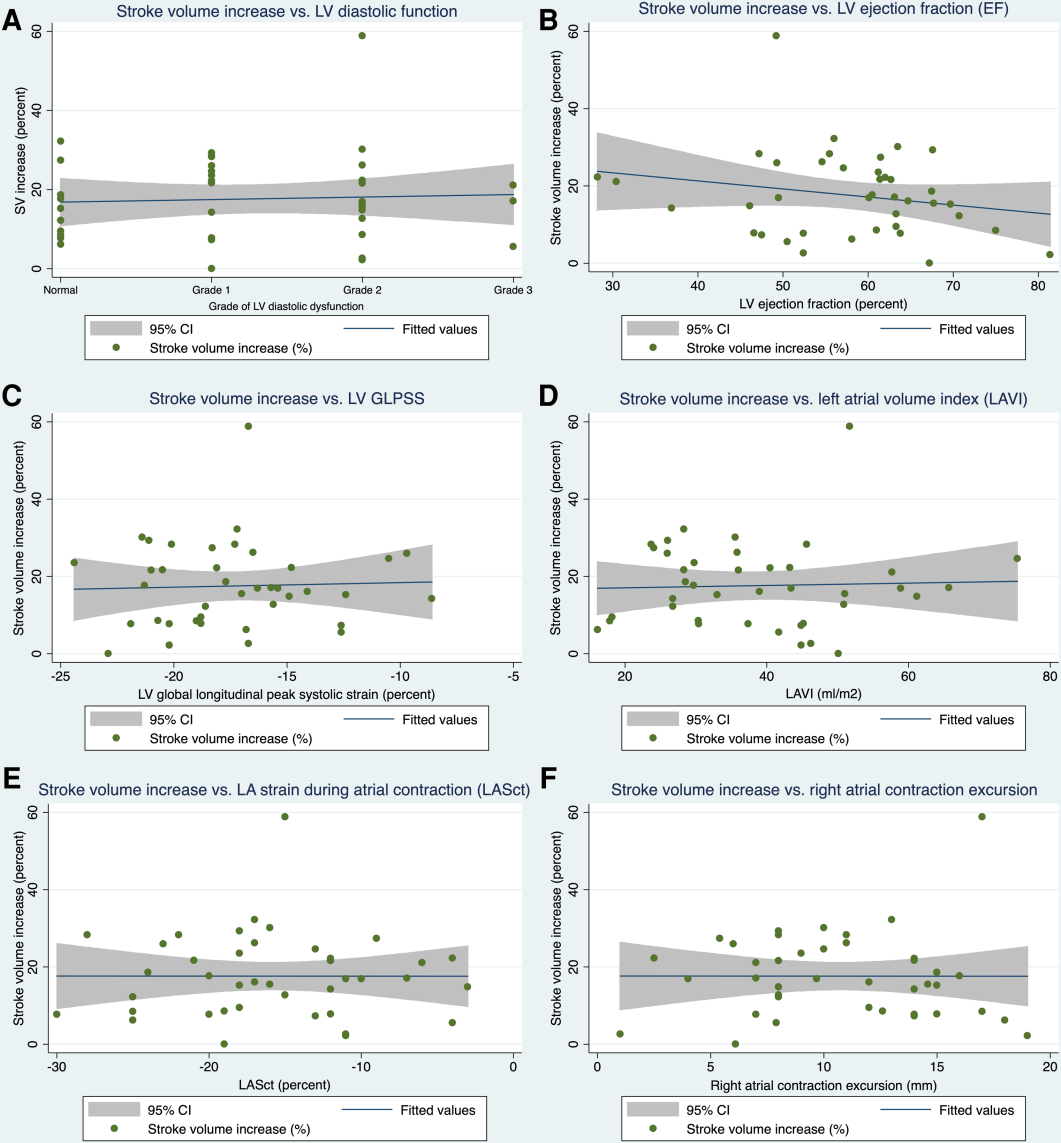
Linear regression models. Association of selected primary (**A**) and secondary (**B–F**) endpoint echo parameters with the increase in stroke volume when pacing in DDD mode as opposed to VVI mode. SV, stroke volume; LV, left ventricular; EF, ejection fraction; GLPSS, global longitudinal peak systolic strain; LAVI, left atrial volume index; LASct, left atrial strain during the contraction phase of the left atrium, 95% CI = 95% confidence interval.

Applying linear regression models, no firm associations could be detected between primary and secondary endpoint echo parameters and the extent of relative stroke volume increase with AV-synchronous DDD pacing compared with VVI mode (see Table 4 and Figure 1). When looking at the epidemiological baseline variables, patients with a history of acute myocardial infarction (AMI) benefited less from DDD pacing in terms of relative stroke volume gain. On an absolute scale, the median stroke volume increase was 14.0 ml (IQR: 7.7 ml–16.0 ml) in 34 patients without a prior AMI and 5.0 ml (IQR: 3.3 ml–7.0 ml) in 6 subjects with a history of AMI. These numbers correspond to a relative increase of 17.4% (IQR: 12.7%–26.0%) and 6.9% (IQR: 5.5%–9.5%), respectively. The association between AMI status and relative stroke volume increase proved robust in a multivariate regression model that adjusted for diastolic and systolic left ventricular function parameters as well as important clinical confounders (Table 5). The primary indication for device implantation (*p* = 0.187) or the RV lead localization (*p* = 0.412) were not associated with the relative stroke volume increase with AV-synchronous DDD pacing.

**Table 4:** Results of univariate linear regression models investigating the association between the relative increase in stroke volume (%) and primary endpoint echo variables (1), secondary endpoint echo variables (2), as well as selected epidemiological baseline variables (3).

Parameter	Regression coefficient	95% CI	*p*-value	Parameter	Regression coefficient	95% CI	*p*-value
**Primary endpoint echo variables**							
Grade of diastolic dysfunction	0.64	−3.21–4.49	0.740	E-wave deceleration time	0.01	−0.04–0.06	0.781
E/A ratio	−0.11	−6.48–6.26	0.973	S-wave velocity	0.17	−0.11–0.44	0.220
E/E’ septal	−0.02	−0.41–0.38	0.933	D-wave velocity	−0.06	−0.29–0.17	0.604
Averaged E/E’	0.04	−0.51–0.58	0.898	S/D ratio	4.05	−4.24–12.35	0.329
**Secondary endpoint echo variables**							
LV EF	−0.21	−0.53–0.11	0.191	LAVI	0.03	−0.23–0.29	0.815
LV GLPSS	0.12	−0.89–1.12	0.814	LA GLPSS	0.02	−0.42–0.46	0.915
LV-EDD	0.04	−0.48–0.56	0.885	LASr	0.01	−0.26–0.28	0.959
LV-EDV	0.03	−0.06–0.12	0.512	LAScd	0.15	−0.48–0.78	0.623
LV-ESD	0.11	−0.31–0.52	0.603	LASct	−0.001	−0.54–0.54	0.996
LV-ESV	0.06	−0.07–0.18	0.390	TAPSE	0.21	−0.56–0.98	0.589
LA volume	0.03	−0.10–0.16	0.673	RACE	−0.003	−0.82–0.82	0.994
**Baseline variables**							
Age	−0.07	−0.39–0.26	0.682	Amount of ventricular pacing	−0.02	−0.89–0.85	0.965
Sex (female)	−4.69	−13.00–3.62	0.260	Optimized AV-delay	0.02	−0.06–0.10	0.670
BMI	−0.31	−1.34–0.72	0.551	Heart rate (DDD/VVI)	−0.18/−0.16	−0.46–0.10/−0.45–0.13	0.201/0.276
BP syst	0.07	−0.10–0.24	0.416	H/o diabetes	−4.14	−11.72–3.44	0.276
BP diast	0.13	−0.11–0.37	0.289	GFR	0.06	−0.12–0.25	0.487
Amount of atrial pacing	−0.003	−0.13–0.12	0.963	H/o AMI	−10.71	−19.94–−1.48	0.024

CI, confidence interval; LV, left ventricular; EF, ejection fraction; GLPSS, global longitudinal peak systolic strain; EDD, end-diastolic diameter; ESD, end-systolic diameter; EDV, end-diastolic volume; ESV, end-systolic volume; LA, left atrial; LAVI, left atrial volume index; LASr, left atrial reservoir strain; LAScd, left atrial conduit strain; LASct, left atrial contraction strain; TAPSE, tricuspid annular plane systolic excursion; RACE, right atrial contraction excursion; BMI, body mass index; BP syst, systolic blood pressure; BP diast, diastolic blood pressure; h/o, history of; GFR, glomerular filtration rate; AMI, acute myocardial infarction.

**Table 5:** Results of multivariate linear regression model investigating the association between the relative increase in stroke volume (%) and selected echo parameters and important clinical confounders.

Parameter	Regression coefficient	95% CI	*p*-value
AMI status	−12.55	−23.79 to −1.31	0.030
LV EF	−0.29	−0.72–0.14	0.182
LV diastolic function	−0.21	−5.89–5.47	0.941
LAVI	−0.08	−0.48–0.33	0.700
LA GLPSS	0.17	−0.40–0.74	0.541
TAPSE	0.11	−0.78–1.00	0.800
Age	−0.03	−0.41–0.34	0.850
Sex	−5.30	−15.91–5.31	0.315
BMI	−0.64	−1.99–0.72	0.344
GFR	0.10	−0.13–0.33	0.872
Diabetes status	0.85	−9.91–11.62	0.872

CI, confidence interval; AMI, acute myocardial infarction; LV, left ventricular; EF, ejection fraction; LA, left atrial; GLPSS, global longitudinal peak systolic strain; LAVI, left atrial volume index; TAPSE, tricuspid annular plane systolic excursion; BMI, body mass index; GFR, glomerular filtration rate.

### 3.4. Sub-analysis for AMI status

Because of the association between AMI status and stroke volume increase, we further examined differences in baseline characteristics and echocardiographic data as a function of AMI status (see Supplementary Tables S1, S2). AMI was diagnosed based on a posterior wall STEMI in 3 cases and on an NSTEMI in the remaining 3 cases. The time between AMI and inclusion in the study ranged from 1 to 36 years. Patients in the AMI group were more likely to be diagnosed with diabetes (67% vs. 24%, *p* = 0.055), have lower systolic and diastolic blood pressure at baseline (median 135 mmHg vs. 158 mmHg, *p* = 0.012, and 73 mmHg vs. 86 mmHg, *p* = 0.092, respectively), and take spironolactone (33% vs. 0%, *p* = 0.019), whereas no difference was observed for other baseline parameters. A-wave and S-wave velocities, both primary endpoint echo parameters used to assess diastolic LV function, were lower in the AMI group, whereas no differences were found for the other echo parameters. The A-wave velocity was median 77.4 cm/s (IQR: 49.0–90.2) in the AMI group and 96.5 cm/s (IQR: 77.6–109.0) in the group without AMI (*p* = 0.056). S-wave velocities were 45.8 cm/s (IQR: 40.4–54.1) and 60.4 cm/s (IQR: 52.9–68.2), respectively (*p* = 0.036). Although average blood pressure values were lower in the AMI group than in patients without previous myocardial infarction, there was a trend toward a steeper increase with DDD pacing - as opposed to VVI pacing. The rise in diastolic blood pressure with DDD pacing was markedly more pronounced in the AMI group (rise in systolic blood pressure: median 7.5 mmHg in the AMI group and 4.5 mmHg in the non-AMI group, *p* = 0.850, rise in diastolic blood pressure: median 9.5 mmHg in the AMI group vs. −1 mmHg in the non-AMI group, *p* = 0.053).

## 4. Discussion

### 4.1. Summary of findings

In our cohort of patients in sinus rhythm, each subject benefited from DDD pacing in terms of stroke volume increase (median: 12.8 ml/16.9%). We also observed a higher blood pressure and a trend towards less severe mitral regurgitation with AV-synchronous DDD pacing. None of the 26 single echo parameters studied could adequately predict the magnitude of stroke volume increase. However, patients with a history of AMI were found to benefit less compared with the other study participants. The AMI group comprised six individuals, four of whom were diabetics. AMI patients had on average lower baseline blood pressure values. In addition, the A- and S-wave velocities were reduced in patients after AMI compared with the rest of the study cohort.

### 4.2. Pathophysiological interpretation of study results

The wide range of stroke volume increase with AV-synchronous DDD pacing compared with AV-asynchronous VVI pacing in our study (increase of 0.1%–58.9%) was also observed in a trial by Masuyama et al. that investigated the same study question in 1986 (increase of 3%–73%) (11). The authors sought to identify parameters affecting the change in cardiac output as a function of pacing mode. In their analysis, impaired LV rapid filling as a parameter of diastolic LV function was predictive of a greater rise in cardiac output. In the MARVEL 2 cohort, atrial strain and E/A-wave ratio were associated with the strength of atrial contraction related accelerometer signals, which can be seen as a proxy for atrial function and the extent of the atria's contribution to ventricular filling (19).

As optimally timed atrial contraction may increase the cardiac output by up to 30% (18), we optimized the AV-delay before measuring stroke volume to eliminate this potential source of bias (20, 21). Applying novel echo parameters and new definitions of LV diastolic function in our study, we did not identify any of the echo parameters mentioned above to be associated with an increase in stroke volume. Furthermore, the location of the RV lead - that might have influenced the ventricular contraction pattern and thus stroke volume - was not associated with the change in stroke volume. We did find, however, that post-AMI patients had a smaller stroke volume increase with AV-synchronous pacing than the rest of the cohort. In these subjects, A-wave and S-wave velocities were reduced compared with the other study participants. A reduced A-wave velocity may be caused by left ventricular dysfunction and subsequent rise in LVEDP, left atrial dysfunction, or a combination of both (18). A decline of S-wave velocity may be due to impaired LV function, right ventricular dysfunction (less blood is pushed through the lungs into the left atrium), or left atrial dysfunction (rise of pressure during atrial contraction is limited) (22–24). A combination and interplay of all these factors in post-AMI patients most likely explains our findings, as no other echo parameters were found to be different between the AMI group and the rest of the cohort. Left atrial conduit strain, an early marker of left ventricular diastolic dysfunction, was numerically slightly lower in the AMI group (see Supplementary Table S2) (25). As opposed to LA strain parameters, that are only assessed in one cut plane, A-wave (and to a lesser degree S-wave) velocity may provide a more integral assessment of left atrial function and pressure conditions. Whereas left ventricular ejection fraction and global longitudinal strain at rest did not differ between patients with and without a history of a previous AMI, we cannot exclude subclinical systolic left ventricular dysfunction as no stress test was performed. In addition, the Frank-Straub-Starling mechanism may be compromised after AMI. Under normal conditions, the increased ventricular filling during AV-synchronous pacing would result in an increased stretch of LV myocardial fibers, which in turn would increase myocardial contractility and thus LV stroke volume. In patients after myocardial infarction, this mechanism might be attenuated, e.g., in smaller regions of scarring and/or cellular dysfunction (26). Furthermore, it is conceivable that the relative contribution of a properly timed atrial contraction to left ventricular filling may differ at different heart rates (force-frequency relationship) and that, after a myocardial infarction, the “optimal” heart rate may be higher or lower than otherwise (27). Baseline heart rate during DDD pacing was virtually the same in the non-AMI and AMI groups (65 and 67 beats per minute, respectively). As we only performed stroke volume measurements at one heart rate (the intrinsic heart rate at the time of the examination), we cannot exclude a possible effect. However, measurements at various heart rates would have mandated atrial pacing for heart rates above the intrinsic sinus rate, and assessment of heart rates below the intrinsic rate would have been impossible. Moreover, pacing the atrium might have altered atrial myocardial mechanics, potentially confounding the results. Furthermore, better contractility at higher heart rates cannot be expected with higher oxygen consumption in coronary artery disease, and a heart rate lower than 60 beats per minute at rest is considered too low in daily life.

### 4.3. Blood pressure and mitral regurgitation

As expected from a physiological point of view, the increase in stroke volume with AV-synchronous pacing tended to increase blood pressure. The rise in diastolic blood pressure with DDD pacing was especially pronounced in the AMI group. We hypothesize that this effect was caused due to higher arterial stiffness in this group (more diabetics and probably a higher atherosclerotic burden after AMI), even with relatively small rises in stroke volume. Reducing mitral regurgitation with AV-synchronous pacing is a well-known phenomenon observed as a trend in our data (28). However, it must be emphasized that assessment of mitral regurgitation was not the focus of this study, and patients with moderate or severe MR were not eligible for inclusion. Thus, interpretation of this finding is substantially limited.

### 4.4. Limitations

This study was a single-center study with all the limitations inherent in such. Because of strict inclusion and exclusion criteria and the complex echocardiographic examination protocol, only 40 participants could be included, which limited the statistical power. The volume status may have been a confounding factor that was not assessed during the echocardiographic examination. In addition, the number of patients with heart failure with reduced ejection fraction (HFrEF, i.e., an LV EF below 40%) was low (3 subjects, 7.5%). Hence, our findings must be interpreted with caution in this subgroup. As this was a hypothesis-generating study, larger multi-center trials with larger sample sizes will be necessary to corroborate our findings.

## 5. Conclusions

In our cohort of patients in sinus rhythm, each subject benefited from AV-synchronous DDD pacing in terms of stroke volume increase. No single echo parameter under investigation could sufficiently predict the amount of stroke volume response. However, we could show that the beneficial effect, while still present, was attenuated after a prior acute myocardial infarction. In patients after an AMI, A- and S-wave velocities were found to be reduced. These results may form the basis for further studies to determine which patients are best suited for a leadless pacemaker in the presence of complete AV block.

## Data Availability

The raw data supporting the conclusions of this article will be made available by the authors, without undue reservation.
