# Supplementary material for: Impact of pacing mode and different echocardiographic parameters on cardiac output (PADIAC)
Source: Front Cardiovasc Med. 2023 May 17;10:1185518. doi: 10.3389/fcvm.2023.1185518 (PMC10231674; doi:10.3389/fcvm.2023.1185518)
Supplement: Supplementary file 1 [file Table1.docx]

Supplementary Material

**Impact of Pacing Mode and Different Echocardiographic Parameters on Cardiac Output (PADIAC)**

**Hermann Blessberger, MD^1,2^*, Juergen Kammler, MD^1,2,3^, Joerg Kellermair, MD, PhD^1,2^, Daniel Kiblboeck, MD^1,2^, Alexander Nahler, MD^1,2^, Denis Hrncic, MD^1,2^, Karim Saleh, MD^1,2^, Stefan Schwarz, MD^1,2^, Christian Reiter, MD^1,2^, Alexander Fellner, MD^1,2^, Christian Eppacher, PhD^4^, Todd J. Sheldon. MSc^5^, Clemens Steinwender, MD^1,2,3^**

^1^ Kepler University Hospital, Department of Cardiology, Linz, Austria

^2^ Johannes Kepler University, Medical Faculty, Linz, Austria

^3^ Department of Internal Medicine II, Paracelsus Medical University, Salzburg, Austria

^4^ Medtronic Austria GmbH, Vienna, Austria

^5^ Medtronic PLC, Mounds View, Minnesota, US

*** Correspondence:**Hermann Blessberger, MD^1,2^

^1^ Kepler University Hospital Linz, Department of Cardiology, Med Campus III, Krankenhausstrasse 9, 4020 Linz, Austria.

^2^ Johannes Kepler University Linz, Medical Faculty, Altenbergerstrasse 69, 4040 Linz, Austria.

Tel.: +43/5/7680/83/6220

Fax: +43/5/7680/83/62-05

e-mail: [hermann.blessberger@kepleruniklinikum.at](mailto:hermann.blessberger@kepleruniklinikum.at)

**Table S1. Baseline parameters as a function of AMI status.**

| **Parameter** | **Median (IQR) or count (%)** | | **P-value** | **Parameter** | **Median (IQR) or count (%)** | | **P-value** |  |
| --- | --- | --- | --- | --- | --- | --- | --- | --- |
|  | **History of AMI (n=6)** | **No history of AMI (n=34)** |  |  | **History of AMI (n=6)** | **No history of AMI (n=34)** |  | |
| Age (years) | 72  (70-78) | 77  (71-81) | 0.425^†^ | ACE inhibitors | 3  (50.0) | 15  (44.1) | 1.000^‡^ | |
| Sex (female) | 0  (0) | 9  (26.5) | 0.306^‡^ | AT_2_-blockers | 2  (33.3) | 8  (23.5) | 0.629^‡^ | |
| BMI (kg/m^2^) | 27.6  (26.8-29.3) | 27.2  (24.0-29.4) | 0.649^†^ | Spironolactone | 2  (33.3) | 0  (0.0) | 0.019^‡^ | |
| BP systolic (mmHg) | 135  (127-142) | 158  (142-167) | 0.012^†^ | Arterial hypertension | 6  (100.0) | 28  (82.4) | 0.565^‡^ | |
| BP diastolic (mmHg) | 73  (69-85) | 86  (74-93) | 0.092^†^ | Diabetes mellitus type II | 4  (66.6) | 8  (23.5) | 0.055^‡^ | |
| Heart rate (bpm) DDD | 67  (50-74) | 65  (59-75) | 0.633^†^ | Congestive heart failure | 2  (33.3) | 7  (20.6) | 0.602^‡^ | |
| Heart rate (bpm) VVI | 61  (50-70) | 65  (60-75) | 0.368^†^ | Hyperchol-esterolemia | 6  (100.0) | 19  (55.9) | 0.067^‡^ | |
| Atrial pacing (%) | 40.5  (25.9-58.0) | 39.7  (16.0-59.0) | 0.622^†^ | Peripheral artery disease | 1  (16.7) | 1  (2.9) | 0.281^‡^ | |
| Ventricular pacing (%) | 99.5  (99.0-100.0) | 99.9  (98.0-100.0) | 0.934^†^ | Stroke | 0  (0.0) | 7  (20.6) | 0.629^‡^ | |
| Beta-blockers | 3  (50.0) | 17  (50.0) | 1.000^‡^ | Chronic kidney disease | 2  (33.3) | 15  (44.1) | 1.000^‡^ | |

**Table S1: Baseline characteristics of the study cohort according to AMI status. Physiological parameters, medication, and prior medical history at the time of echo examination. IQR = interquartile range, BMI = Body Mass Index, bpm = beats per minute, BP = blood pressure. ^†^Mann-Whitney U test, ^‡^Chi-square test with Fisher’s exact modification.**

**Table S2. Echocardiographic parameters as a function of AMI status.**

| **Parameter** | **Median (IQR) or count (%)** | | **P-value** |
| --- | --- | --- | --- |
|  | **History of AMI (n=6)** | **No history of AMI (n=34)** |  |
| SV increase with DDD pacing (ml) | 5.0 (3.3-7.0) | 14.0 (7.7-16.0) | 0.031^†^ |
| Relative SV increase with DDD pacing (%) | 6.9 (5.5-9.5) | 17.4 (12.7-26.0) | 0.014^†^ |
| Image quality | 2.5 (2-3) | 2.0 (1-2) | 0.441^‡^ |
| Optimized AV-delay (ms) | 105 (75-160) | 97.5 (80-120) | 0.893^†^ |
| E-wave (cm/sec) | 63.8 (48.5-76.2) | 74.0 (61.1-98.0) | 0.405^†^ |
| A-wave (cm/sec) | 77.4 (49.0-90.2) | 96.5 (77.6-109.0) | 0.056^†^ |
| E-wave deceleration time (ms) | 289 (275-296) | 268 (250-320) | 0.705^†^ |
| E/A ratio | 0.7 (0.7-1.6) | 0.8 (0.6-1.2) | 0.618^†^ |
| E’ septal | 4.4 (3.8-5.6) | 4.5 (3.8-5.3) | 0.955^†^ |
| E’ lateral | 5.8 (5.4-6.7) | 6.6 (5.4-9.8) | 0.297^†^ |
| E/E’ septal ratio | 13.2 (11.3-17.6) | 16.6 (13.0-19.2) | 0.449^†^ |
| Averaged E/E’ ratio | 11.8 (11.3-16.0) | 14.2 (10.7-16.2) | 0.557^†^ |
| S-wave (cm/sec) | 45.8 (40.4-54.1) | 60.4 (52.9-68.2) | 0.036^†^ |
| D-wave (cm/sec) | 40.0 (37.9-65.5) | 48.5 (38.3-64.3) | 0.834^†^ |
| S/D ratio | 2.6 (2.0-2.7) | 2.7 (2.5-2.9) | 0.076^†^ |
| Tricuspid regurgitation velocity (m/sec) | 2.6 (2.0-2.7) | 2.7 (2.5-2.9) | 0.166^†^ |
| Diastolic dysfunction (grade) | 1.5 (1-3) | 2.0 (2-3) | 0.248^‡^ |
| LV-EDD (mm) | 46 (42-52) | 47 (42-49) | 0.703^†^ |
| LV-ESD (mm) | 31 (28-48) | 33 (28-37) | 0.805^†^ |
| LV-EDV (ml) | 83.0 (62.0-151.8) | 98.3 (73.0-123.0) | 0.762^†^ |
| LV-ESV (ml) | 32.3 (21.1-76.7) | 39.3 (25.0-59.9) | 0.970^†^ |
| Septum (mm) | 16 (14-16) | 15 (13-17) | 0.797^†^ |
| Ejection fraction (%) | 60.7 (50.5-63.8) | 60.8 (49.5-63.5) | 0.985^†^ |
| GLPSS (%) | -17.8 (-20.2 to -14.8) | -17.3 (-20.2 to -15.6) | 0.892^†^ |
| LA volume (ml) | 72.9 (44.3-89.7) | 71.9 (54.8-94.9) | 0.545^†^ |
| LAVI (ml/m^2^) | 36.1 (18.2-43.3) | 36.7 (28.3-46.2) | 0.405^†^ |
| LA GLPSS (%) | 25.9 (11.4-27.8) | 23.1 (15.6-26.7) | 0.846^†^ |
| LA reservoir strain (%) | 30 (13-38) | 30 (23-39) | 0.609^†^ |
| LA conduit strain (%) | -11 (-13 to -9) | -13 (-17 to -10) | 0.494^†^ |
| LA contraction strain (%) | -19 (-20 to -4) | -16 (-20 to -12) | 0.894^†^ |
| TAPSE (mm) | 24 (18-27) | 24 (20-28) | 0.543^†^ |
| RACE (mm) | 8 (6-12) | 11 (8-14) | 0.190^†^ |
| BP syst DDD (mmHg) | 127 (120-138) | 145 (137-160) | 0.024^†^ |
| BP syst VVI (mmHg) | 123 (116-134) | 138 (125-154) | 0.056^†^ |
| BP diast DDD (mmHg) | 64 (59-73) | 80 (70-84) | 0.084^†^ |
| BP diast VVI (mmHg) | 61 (46-69) | 73 (66-88) | 0.022^†^ |
| Syst BP increase with DDD pacing (mmHg) | 7.5 (-8 to 11) | 4.5 (-8 to 19) | 0.850^†^ |
| Diast BP increase with DDD pacing (mmHg) | 9.5 (4 to13) | -1 (-5 to 3) | 0.053^†^ |

**Table S2: Echocardiographic parameters according to AMI status. Diastolic dysfunction grade 0 denotes normal diastolic function. LV = left ventricular, LA = left atrial, EDD = end-diastolic diameter, EDV = end-diastolic volume, ESD = end-systolic diameter, ESV = end-systolic volume, GLPSS = left ventricular global longitudinal peak systolic strain, TAPSE = tricuspid annulus plane systolic excursion, RACE = right atrial contraction excursion, BP = blood pressure, syst = systolic, diast = diastolic, DDD = measured in DDD-mode, VVI = measured in VVI-mode. ^†^Mann-Whitney U test, ^‡^Chi-square test with Fisher’s exact modification.**

**Figure S1.**

**
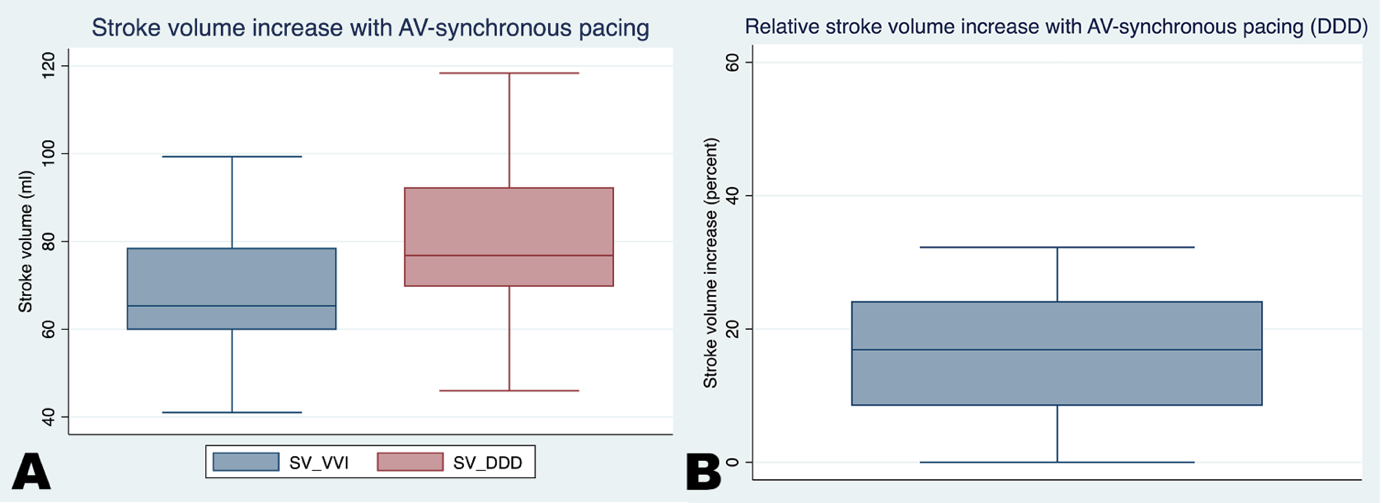
**

**Figure S1: Box and whisker plots depicting (A) the absolute stroke volumes with both pacing modes and (B) the relative increase of left ventricular stroke volume with AV-synchronous DDD pacing as compared with AV-asynchronous VVI pacing. Boxes represent the 25^th^ and 75^th^ percentiles with the median as a solid center line. Whiskers indicate the most extreme values within 1.5 times the interquartile range above the 75th percentile and below the 25th percentile, respectively. SV_VVI = left ventricular stroke volume in VVI pacing mode, SV_DDD = left ventricular stroke volume in DDD pacing mode.**
